# Severe Prolonged Hypocalcemia Following Four-Gland Parathyroidectomy in a Patient With Established Renal Failure

**DOI:** 10.7759/cureus.67964

**Published:** 2024-08-27

**Authors:** Rafid Mustafa, Hosna Ara Begum, Sathia Narayanan Mannath, Cornelius Fernandez James

**Affiliations:** 1 Acute Medicine, Pilgrim Hospital, Boston, GBR; 2 Diabetes and Endocrinology, Pilgrim Hospital, United Lincolnshire Hospitals NHS Trust, Boston, GBR; 3 Endocrinology and Metabolism, Pilgrim Hospital, Boston, GBR

**Keywords:** four-gland parathyroidectomy, established renal failure, hypocalcaemia, hypoparathyroidism, hungry bone syndrome

## Abstract

Hypocalcemia after parathyroidectomy is a common complication. It is typically transient in patients with mild parathyroid-related bone disease. Distinguishing between hungry bone syndrome (HBS) and hypoparathyroidism following parathyroidectomy in established renal failure (ERF) patients presents a significant diagnostic challenge. This case study describes a 44-year-old male with severe hypocalcemia following a four-gland parathyroidectomy, highlighting the diagnostic considerations and management strategies.

## Introduction

Following parathyroidectomy, hypocalcemia can arise from either hungry bone syndrome (HBS) or hypoparathyroidism. HBS is a rare complication that can occur after a successful parathyroidectomy for patients with severe primary hyperparathyroidism, a condition linked to high preoperative bone turnover. HBS is characterized by a sustained and profound decrease in serum calcium (below 2.1 mmol/L or 8.4 mg/dL) levels, which may last up to a year, often accompanied by reduced phosphate and magnesium levels associated with normal or raised parathyroid hormone (PTH) levels. Moreover, HBS patients would have reduced urinary calcium and phosphate excretion [1]. Conversely, hypoparathyroidism is a relatively rare condition. This is mostly seen after parathyroid exploration, total thyroidectomy, or during a repeat operation for thyroid or parathyroid conditions. This condition typically results from the inadvertent removal of the parathyroid glands or its damage, often because of direct injury or impaired blood supply. Hypoparathyroidism presents with hypocalcemia, raised serum phosphate, and low PTH levels [2]. These distinct diagnostic criteria assist in differentiating between the two conditions, aiding appropriate management.

## Case presentation

A 44-year-old man with established renal failure (ERF) came to the emergency department because of significant symptomatic hypocalcemia. He had a history of tertiary hyperparathyroidism and had undergone bilateral neck exploration and four-gland parathyroidectomy without autotransplantation, which occurred 14 days prior (18/02/2023 and 03/03/2023) to this admission. As per histology reports, the parathyroid glands were as follows: the left lower parathyroid gland 15 x 14 x 4 mm and 0.5 g; the left upper parathyroid gland 23 x 17 x 13 mm and 2.7 g; the right lower parathyroid gland 10 x 10 x 8 mm and 0.5 g; and the right upper parathyroid gland 23 x 15 x 13 mm and 2.2 g. He was feeling generally unwell with muscle cramps, tingling, and numbness in his fingers and arms. Trousseau's and Chvostek's signs were positive.

His past medical history includes IgA nephropathy, ERF, renal transplant in August 2022, New Onset Diabetes After Transplant (NODAT) in December 2022, tertiary hyperparathyroidism treated with surgery in February 2023, ulcerative colitis (UC), and previous myocardial infarction. He was taking tacrolimus, mycophenolate, prednisolone, and a proton pump inhibitor (PPI). Before the surgery, he was on cinacalcet, but post-surgery, he was started on alfacalcidol 4 mcg twice daily (BD). On admission, he had hypocalcemia, hyperphosphatemia, and hypomagnesaemia. There was no evidence to suggest gastrointestinal loss of electrolytes. His eGFR was 32 mL/min, consistent with his baseline post-transplant, with a creatinine level of 210 µmol/L and urea of 13.4 mmol/L.

The patient received intravenous calcium and magnesium replacement therapy while continuing alfacalcidol at 4 mcg twice daily. Additionally, oral calcium, cholecalciferol, and magnesium supplements were initiated. Although there was an initial improvement in calcium levels with both intravenous and oral calcium supplementation, a significant drop in calcium levels occurred after the intravenous calcium was discontinued on day five. This decline necessitated the reintroduction of intravenous calcium to maintain adequate serum calcium levels. Despite these interventions, it took nearly eight days to normalize calcium levels and 11 days to normalize magnesium levels.

**Figure 1:**
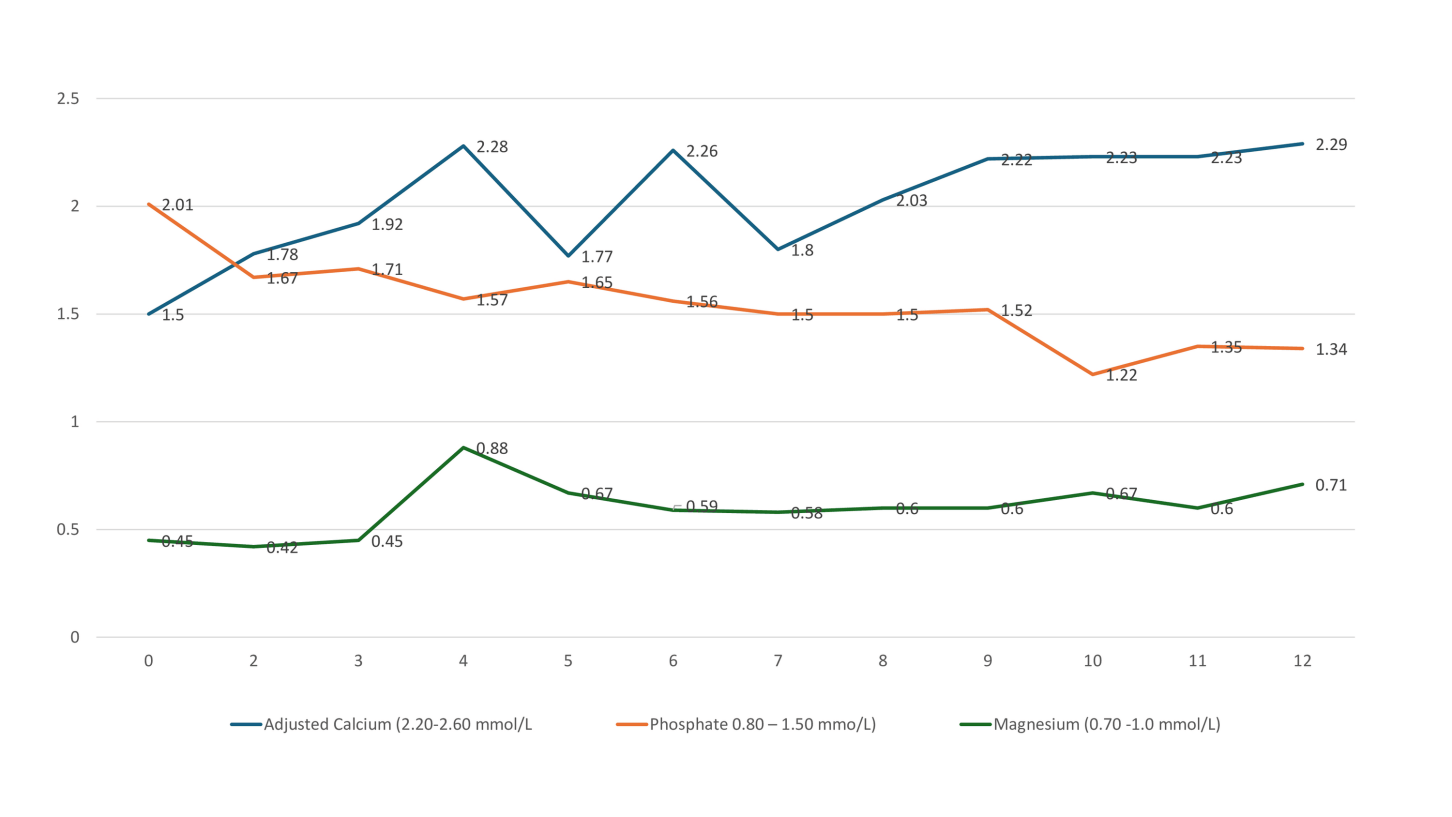
Investigations from day 0 to day 12

The endocrine team initially suspected HBS in this patient, who had a history of tertiary hyperparathyroidism and presented with severe hypocalcemia following a parathyroidectomy. HBS was considered more likely than hypoparathyroidism, as it is a more common complication after such surgeries and is also known to cause profound and prolonged hypocalcemia. Additionally, HBS often leads to hypomagnesemia, a condition not typically associated with hypoparathyroidism. The hypomagnesemia in this case could have also been potentially exacerbated using PPIs and/or tacrolimus [3]. They advised to check 25-hydroxy vitamin D, PTH, and serum tacrolimus trough level 12 hours post-dose); to increase the calcium supplementation to a total daily dose of 46 grams of elemental calcium/day; to ensure that calcium supplementation is provided between meals to increase its absorption; to add additional cholecalciferol based on 25-hydroxy vitamin D levels on top of alfacalcidol; to swap PPI to famotidine; and to swap magnesium aspartate to magnesium glycerophosphate (Table 1, Figure 1).

**Table 1:** Investigations

Day	Adjusted calcium (2.20-2.60 mmol/L)	Phosphate 0.80–1.50 mmol/L)	Magnesium (0.701.0 mmol/L)	Parathyroid hormone (1.6–6.9 pmol/L)	25 hydroxy Vit D (nmol/L)	Tacrolimus (5.0-15.0 mcg/L)
0	1.50	2.01	0.45	-	-	-
2	1.78	1.67	0.42	-	-	-
3	1.92	1.71	0.45	-	-	-
4	2.28	1.57	0.88	-	33	-
5	1.77	1.65	0.67	< 0.2	32	-
6	2.26	1.56	0.59	-	-	-
7	1.80	1.50	0.58	-	-	3.7
8	2.03	1.50	0.60	-	-	-
9	2.22	1.52	0.60	-	-	-
10	2.23	1.22	0.67	-	-	-
11	2.23	1.35	0.60	-	-	-
12	2.29	1.34	0.71	-	-	-

Initial replacement with Adcal-D3 two tablets three times daily (TDS), provided elemental calcium 3.6 grams and cholecalciferol 2,400 units. This was later changed to two tablets four times daily (QDS), which provided 4.8 grams and 3,200 units, respectively. The magnesium aspartate one sachet BD provided 20 mmol of magnesium/day. This was later swapped to magnesium glycerophosphate two tablet TDS, which provided 24 mmol of magnesium/day, with a lesser risk of causing diarrhea. Once PTH came back as low, the endocrine team revised their diagnosis to postoperative hypoparathyroidism.

After 12 days of hospital stay, his calcium and magnesium levels stabilized, and he was discharged home with a revised plan regarding calcium, vitamin D, and magnesium supplementation (alfacalcidol 4 mcg BD, Adcal-2 tablet TDS, cholecalciferol 1,000 units once daily (OD), magnesium glycerophosphate two tablets TDS) and outpatient follow-up with weekly calcium, phosphate, and magnesium monitoring and follow-up.

## Discussion

Initially, we suspected HBS in this patient, who presented with severe hypocalcemia following parathyroidectomy, given his history of tertiary hyperparathyroidism and renal transplantation. HBS is a more common complication than hypoparathyroidism and is known to cause profound hypocalcemia along with hypomagnesemia [1]. Since hypomagnesemia is not typically seen in hypoparathyroidism, other potential causes, such as PPI or tacrolimus-induced hypomagnesemia [3], should have been considered alongside hypoparathyroidism. For a diagnosis of HBS, PTH levels should be normal or high (although lower than preoperative levels) with low phosphate levels. In contrast, patients with hypoparathyroidism would exhibit lower-than-normal PTH levels, along with high normal or elevated phosphate levels [2]. Diagnosing HBS in patients with primary hyperparathyroidism is relatively straightforward, but it becomes more challenging in those with tertiary hyperparathyroidism, as the latter is associated with hyperphosphatemia. While the immediate management of acute hypocalcemia is similar in both HBS and hypoparathyroidism, differentiating between the two is crucial for prognosis. HBS is a more serious condition, often associated with prolonged hypocalcemia, significant morbidity, and extended hospitalization, whereas hypoparathyroidism is typically transient.

HBS

HBS involves prolonged hypocalcemia after parathyroidectomy for primary and secondary/tertiary hyperparathyroidism, especially in patients with ERF. HBS can also occur after thyroidectomy for thyrotoxicosis and in metastatic prostate cancer, the latter because of a sudden shift to osteoblastic activity after high bone turnover [1,4,5].

Various risk factors including raised levels of PTH, blood urea nitrogen, and alkaline phosphatase, raised body mass index, and enhanced gland size correlate with the development of HBS. Bone diseases like brown tumors, fractures, and osteitis fibrosa cystica along with high osteoclast numbers on biopsy also increase the risk for HBS. In primary hyperparathyroidism, older age and high preoperative calcium levels raise the incidence. Conversely, in secondary hyperparathyroidism, younger age and lower preoperative calcium levels contribute to it [1]. In our case, with a history of tertiary hyperparathyroidism on a background of renal transplantation, the patient was already on alfacalcidol and cinacalcet before the planned parathyroidectomy in an attempt to mitigate the risk of HBS.

HBS frequency varies among studies. It affects 4-13% after parathyroidectomy for primary hyperparathyroidism and 20-70% after surgery for secondary/tertiary hyperparathyroidism [1].

Patients with HBS may show symptoms of hypocalcemia such as seizures, tetany, numbness, tingling, carpopedal spasms, arrhythmias, cardiomyopathy, and laryngospasm. Physical signs can include fractures, deformities, surgical scars, and positive Trousseau or Chvostek signs [1,6,7].

The treatment for HBS typically involves administering 612 g of elemental calcium/day. When serum calcium levels fall below 1.9 mmol/L 7.6 mg/dL, or if the patient is symptomatic or has electrocardiogram (ECG) changes (prolonged QTc), immediate IV calcium treatment is necessary. Calcium gluconate is preferred over calcium chloride as it is less irritating to tissues and does not require a central line [1,8,9].

Start with a bolus of 10% calcium gluconate, 10 to 20 mL diluted in 50 to 100 mL of dextrose 5% in water (D5W), given over five to 10 minutes. This provides 100 to 200 mg of elemental calcium. This is to be followed by a continuous infusion of 100 mL of 10% calcium gluconate in 1 L of D5W, starting at 50 mL/hour and adjusting every five to six hours based on calcium, phosphorus, and magnesium levels. The treatment goal is to infuse at a rate of 0.5 to 1.5 mg of elemental calcium/kg/hour. Begin oral calcium supplements when the patient can tolerate them [8].

For oral supplementation, calcium carbonate is often preferred because of its higher elemental calcium content 400 mg/g) compared to calcium citrate 211 mg/g). For patients on PPI or with achlorhydria, calcium citrate is preferred over calcium carbonate because of its superior absorption [1,9]. Magnesium should be replenished as needed to aid calcium absorption, but hypophosphatemia should not be aggressively treated to avoid further lowering calcium. Active vitamin D including alfacalcidol 0.5 4 mcg OD or calcitriol 0.254 mcg/day in one or two divided doses) should also be given to improve calcium levels, although its effects will take a few days to become apparent [10].

Hypoparathyroidism

Hypoparathyroidism is characterized by hypocalcemia, elevated serum phosphate, and low or inappropriately normal PTH levels [2,11]. Hypoparathyroidism is commonly caused by neck surgery, with autoimmune diseases as the second leading cause. Disorders of magnesium metabolism can mimic hypoparathyroidism (i.e., hypomagnesemia can cause functional hypoparathyroidism). Rarely, it can result from gland infiltration or genetic disorders. Genetic forms include syndromic and isolated forms, such as autosomal dominant hypocalcemia. Genetic testing is considered in cases with specific indications such as young age or family history [12,13].

The onset of hypocalcemia in hypoparathyroidism may occur acutely following neck surgery or because of changes in calcium and vitamin D supplementation. These acute episodes can manifest as seizures or life-threatening laryngospasm. However, most cases present with chronic symptoms from long-term hypocalcemia and hyperphosphatemia, affecting various organ systems, particularly neurological, cognitive, muscular, and cardiac systems. Chronic hypocalcemia and hyperphosphatemia can also lead to soft tissue calcifications, commonly observed in the brain and kidneys, as well as in joints, eyes, skin, and blood vessels [13].

In hypoparathyroidism, symptomatic hypocalcemia (including carpopedal spasm, seizures, or broncho- or laryngospasm) can necessitate immediate intravenous calcium treatment, constituting a medical emergency. While a corrected serum calcium level below 1.9 mmol/L 7.5 mg/dL often prompts acute management, the presence of symptoms primarily guides the treatment initiation. The approach to managing hypocalcemia in hypoparathyroidism involves calcium replacement, similar to the management of hypocalcemia in HBS. ECG monitoring is advisable when the patient is on digoxin therapy. Additionally, if magnesium deficiency coexists, correcting it is essential alongside calcium administration [13,14].

## Conclusions

Initially, our patient presented with hypocalcemia, hypomagnesemia, and hyperphosphatemia, along with a recent history of four-gland parathyroidectomy. We initially managed the patient under the presumption of HBS. However, upon receiving the PTH results, our diagnosis was revised to hypoparathyroidism. The hypomagnesemia was attributed to PPI or tacrolimus use. Consequently, the tacrolimus level returned normal, suggesting that PPI was the probable cause of hypomagnesemia. We tailored our treatment approach, accordingly, focusing on managing hypoparathyroidism, and, upon discharge, the electrolyte levels returned to normal.

In conclusion, distinguishing between HBS and hypoparathyroidism in patients with hypocalcemia after parathyroidectomy is crucial for effective management. HBS is characterized by prolonged low calcium, low phosphate, and low magnesium levels with normal or raised PTH. Conversely, hypoparathyroidism features low calcium, high serum phosphate, and low PTH levels. Recognizing these distinct biochemical profiles allows for a systematic, diagnostic, and treatment approach, improving patient outcomes and minimizing complications.
